# Unforeseen enemy: African swine fever

**DOI:** 10.5713/ajas.2020.0001ED

**Published:** 2019-12-23

**Authors:** Cheol-Heui Yun

**Affiliations:** 1 Department of Agricultural Biotechnology, College of Agriculture and Life Sciences, Seoul National University, Seoul 08826, Korea; 2 Co-Editor-in-Chief, Asian-Australasian Journal of Animal Sciences, Seoul 08776, Korea

African swine fever (ASF) is a severe viral disease that affects domestic and wild pigs and causes major production and economic losses. Many ASF virus (ASFV) strains cause the death in almost 100% of the infected pigs. In May 24, 2004, the Global Framework for the Progressive Control of Transboundary Animal Diseases (GF-TADs), called “FAO-OIE GF-TADs general agreement”, was launched. GF-TADs is a joint initiative of FAO and OIE, with the participation of the World Health Organization for zoonoses, and its goals are to achieve the prevention, detection, and control of TADs by focusing their original and global dimensions [1].

Historically, ASF outbreaks were mainly reported in Africa, parts of Europe, and South America. The disease seemingly spread in multiple countries across Africa, Asia, and Europe in both domestic and wild pigs. A recent outbreak of ASF has swept across Asian countries. The spread of the disease is more than a cross-boundary problem, and, everybody has to pitch in against the deadly ASFV. In China, 163 cases were reported in 32 provinces, and about 1,192,000 pigs have been culled since the first ASF outbreak in Liaoning Province on August 3, 2018. As shown in Figure 1, ASF spread rapidly through other Asian countries. In 2019, it spread to Mongolia (January), followed by Vietnam (February); Cambodia (April); Hong Kong and North Korea (May); Laos (June); Myanmar (August); and the Philippines, South Korea, and Timor-Leste (September). The ASF outbreak in Vietnam was serious, as it has been spreading rapidly since the first outbreak on February 19, 2019. The Food and Agriculture Organization (FAO) of the United Nations found that a total of 62 provinces and cities have reported outbreaks, and about 3.7 million pigs have either died from the disease or been culled as a control measure. According to the FAO, almost 5 million pigs in Asia have died or been culled because of ASF as of August 2019 [1].

The risk of ASFV mutating to become infectious to humans is extremely low; however, the economic cost of an epidemic and/or endemic will be enormous without counting the invisible cost. The death of approximately 100,000 pigs in only 2 months after the virus was first detected cost the Chinese economy US$ 20 million [2]. In China, pork price skyrocketed to 46.7% year-on-year in August after rising by 27% in July this year. China has roughly 440 million pigs, and future ASF epidemics will spell disaster for not only China but also the rest of the world. According to Nature News [3], China has invested US$ 15 million in research on ASFV, encouraging researchers to fill many gaps in our understanding of the virus, including its detailed structure and how it transmits to hosts and evades host immune systems. ASF killed as much as a third of the pig population in China by August 2019. Thus, government officials are discussing dramatic measures to stabilize the world’s largest pork market. The high prices would likely lead to pressure on China to import more pork from other countries. Rabobank estimated that China’s pork production could decrease by 25% this year, suggesting that as much as 1.5 million metric tons of pig carcasses need to be imported. Rabobank expects a 25% decrease in pork meat output in China in 2019 because of ASF, and the pork supply will struggle to recover over the next few years. In fact, China’s pork import from Europe has increased by 54% in the first half of 2019, mostly from Spain, Germany, Denmark, the Netherlands, and France. Interestingly, the outbreak was associated with worries about heparin shortage because the active ingredient of heparin is extracted from the mucous membrane of pig intestines and China is responsible for about 80% of the heparin produced worldwide [4]. The irony is that if the spread of ASFV and culling of infected pigs continue in China, then the world supply of heparin will be threatened.

The main reasons for the unprecedented and continuous spread of ASF are trade activities and continuous movement of infected wild-boar populations among the regions. Particularly, there is still no approved vaccine against ASF, unlike the classical swine fever or hog cholera (which is caused by a different virus). Although there have been claims to present an attenuated vaccine for ASF at the symposium on swine fever in Beijing, we must keep in mind the disaster that occurred when a large number of pigs in Portugal and Spain were vaccinated with an attenuated vaccine in a field trial in the 1960s.

It is perhaps the time for us to move towards finding possible solutions by dissecting the roles of national and international organizations in conjunction with research institutes and private sector. Sanitation is the best practice to prevent any diseases, including ASF; however, control of access to feed, personnel, and vehicles as well as unexpected intruders like wild boar and birds have limitations. ASF, a TAD, can be spread by live or dead pigs, domestic or wild, and pork products. FAO-OIE provided a recent insight on ASF in wild boar. Furthermore, transmission can occur via contaminated feed and fomites such as shoes, clothes, vehicles, knives, equipment, etc., because of the high environmental resistance of ASFV. In farm areas, producers must strictly follow regulations and ensure that an appropriate vaccination schedule is maintained. Furthermore, producers should be actively involved in setting up an alarming system for disease occurrence. In research institutes, scientists should focus on the mechanism and function of ASFV and define invasion, pathogenesis, immune evasion, host-microbe interaction, and development of vaccines and treatment.

Governments and international organizations should formulate policies and regulations on the basis of the requirement and include research institutes, private sector, and field workers. Of course they should set the guidelines and recommendations, similar to that of FAO [5]. A long-term plan would be the key to resolve disease problems because once the disease outbreak has occurred, then it is extremely hard to eradicate. Therefore, governments and international organizations must set up a proper monitoring system with good intra- and inter-networking among organizations. Furthermore, they should support the need for other organizations, research institutes, and private sector. Sometimes, strong regulations and compliance are necessary, as observed by the actions taken by the Philippines. The Philippines implemented the globally-accepted “1-7-10” protocol to manage, contain, and control the spread of the disease (i.e., all pigs within 1-km radius of infected farms will be culled; limit animal movement; swine farms will be under strict surveillance and testing within a 7-km radius; and swine farms within a 10-km radius will be required to submit a mandatory report on the disease). Zoning was discussed to divide the country into five zones: free zone, containment zone, protected zone, surveillance zone, and infected zone. The infected zone will only be able to trade within their area and Metro Manila with proper documentation [6]. The Department of Agriculture of the Philippines (DA) reiterated the following to its citizens: i) Small backyard raisers should report to their city, municipal, or provincial veterinarian for any sign of disease or death of pigs. Refrain from swill feeding, particularly those from airlines, hotels, and restaurants. Do not slaughter sick or dead pigs and sell the meat to traders. ii) Traders should not buy and slaughter sick pigs. Any backyard or illegal slaughtering or use of meat from infected pigs will contribute to the spread of the disease and will result in further deaths and losses to the 260-billion-Peso (i.e., about US$ 5,139,310,800.00) swine industry that supports millions of Filipinos. iii) For the public: pork is safe to eat. When buying pork in the market or meat shops, always look for the seal and certificate issued by the DA-National Meat Inspection Service.

National considerations and inter-regional collaboration and solidarity are required for strengthening intra-regional networks on disease management and diagnostic protocols. Furthermore, pig and pork value chains within a country and in neighboring countries should be analyzed to improve risk management. One of the most recent (November 4–8, 2019), yet important, actions taken by the FAO has been “the Regional Laboratory Coordinator undertook a mission to the Democratic People’s Republic of Korea to provide laboratory equipment, reagents and training on ASF diagnostic methodologies” [5].

Emerging virus diseases have been, and will be, a major threat to the health of humans as well as economically important animals. Currently, producers, private sector, government officials, and even research scientists are having a difficult time because the citizens are strongly pushing them to find a solution for the ASF issue. It is needless to say that, first, we need to understand the protective immune system of the host against ASFV in terms of pathogen factors, such as pathogenesis, invasion, and, importantly, evasion; host factors such as antigen-specific immunity (humoral and cellular immune responses); and ASFV-host interaction. Furthermore, we immediately require more than one decent vaccine with acceptable efficacy against ASF. We should all work hard before it is too late to overcome this difficult challenge via national and international networks together with useful information, most of which are provided by EMPRES-AH [5].

## Figures and Tables

**Figure 1:**
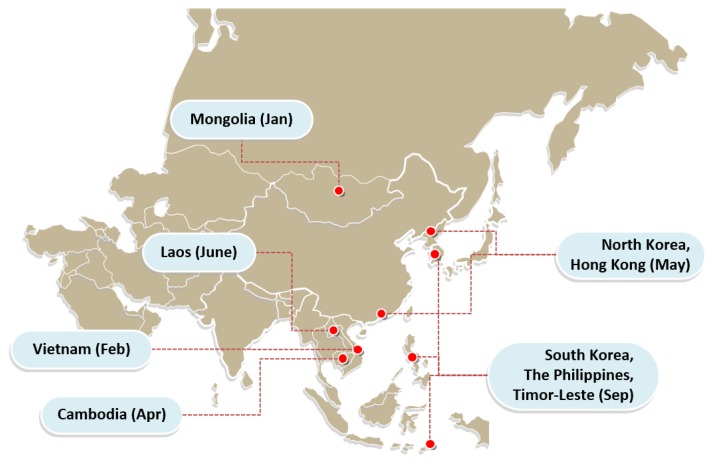
African swine fever outbreaks in Asia in 2019.
